# Chloroformization in Midwifery

**Published:** 1857-05

**Authors:** J. N. Graham


THE NORTH-WESTERN

MEDICAL AND SURGICAL JOURNAL.

(NEW SERIES.)

VOL. VI. MAY, 1857. NO. 5.

### ORIGINAL COMMUNICATIONS.

ARTICLE I.—*Chloroformization in Midwifery.* By J. N. GRAHAM, M.D.

PROF. N. S. DAVIS:

Though a stranger to your columns, it may not be improper to ask your indulgence in a few words on the above topic from my own experience.

Though the article in the main has been published from my pen in a Southern medical journal, some of the facts in the case may not have occurred to the minds of all the readers of your excellent periodical; and I am aware that even now, after all the light that has been thrown upon this subject, it is regarded by many with feelings of distrust—of *horror*. There are not wanting, even now, in our own profession, I am sorry to say, those who regard the defenders of the use of chloroform, as an anæsthetic in child-birth, as wild innovators—*reckless experimenters.*

Men naturally love the old beaten track, and any innovation upon this, whether from empiricism or the result of scientific research, often meets with bitter opposition from those from whom we might expect better things. But it should not weaken our courage or lessen our faith in aught that has truth for its basis, to find the popular clamor raised against; or to find those who, schooled in the notions of the past, are either too lazy to investigate for themselves, or too superstitious to give credence to the results of the investigations of others, look with a holy horror upon anything outside the visual angle of their own limited views. Like the seer of Judah's warrior, pointing with their dismal wand, and from the profundity of their untold knowledge, warning us with holy awe, that 'this portends of *evil* and *only evil.*' Or, like the wizard of Scotia's chief, assume the teachings of the Holy, and tell us,

> ' 'Tis the sunset of life gives them mystical lore:
> And coming events cast their shadows before.'

With solemn look and meaning shake of the head, they would warn us of the 'awful danger of these reckless experiments,' or, as if at once to silence all further research or trial of so potent an agent, they open to our astonished visions, with the sanctity of the inspired upon their lips, the pages of Holy Writ, and tell us 'In sorrow shalt thou bring forth.' Now, we have simply to say here that we are *out* on all such reasoning. Wherever indulged in, it is a disgrace to the profession, and a shame to the man who utters it. In part, this kind of reasoning might have been pardonable years ago, but not now.

I speak thus plainly, because, for more than eight years past, I have witnessed much of this manner of meeting this subject; this unphilosophical method of treating the question of chloroform, as an anæsthetic in child-birth.

Experience is the best guide in all matters that require proof. When we have touched, tasted, and handled for ourselves, then, and not till then, are we capable of thoroughly understanding, or of forming a reliable opinion; it matters not, what may have been our erudition on other points. We need not stop here to give the origin or history of chloroform; this, to all who have given any attention to the subject, must be familiar. Suffice it to say, its powers of abolishing pain were discovered by actual experiment. Its introduction was philosophical, and the accumulating proof in its behalf, as a safe and sure anæsthetic, places it beyond a shadow of doubt, nay, forces us to rank it among other important and unquestionable scientific discoveries.

In our own practice, for the last eight years, we have administered chloroform by inhalation in over one thousand cases, and without an unfavorable result in a single case. We have given it by the mouth for various diseases, such as neuralgia, colic, hysteria, etc. in a very large number of instances, and with the most pleasing results. In three hundred obstetric cases of which we have kept record, with special reference to the time, effects, results, &c. we have administered chloroform in two hundred and thirteen cases, and with the happiest results. It no longer admits of a doubt, that chloroform can be given in child-birth, with perfect success; that it does allay the suffering of parturition, and, that, without disturbing any of its natural functions.

We shall not stop, now, to discuss the results of this agent pathologically (for, as yet, this may be a mere matter of opinion or speculation): whether, like poisonous narcotics or other agents, it may be supposed to affect directly, through the blood, the various ganglia, in an inverse direction to their relative importance to animal life—as, first, the cerebral or intellectual, and, last, the ganglia which support the organic system—thus destroying not only sensation, but all muscular action, and when pushed to the extent, producing death. Be the pathological laws that govern the action of this mysterious agent upon the human system what they may, the results of its wondrous powers over the nervous functions, admit of no doubt—are tangible.

When first inhaled, chloroform produces a sensation described 'as prickling' or 'tingling,' 'like that of a limb asleep.' The patient may complain of a 'roaring noise in her ears;' sometimes the sounds are pleasant, and she likens them to any music that, in consciousness, may be most agreeable to her. Dizziness, or rather confusion, or want of power to *will*, comes on until the patient is lost to all perception of surrounding objects, or lives in a fairy world of her own imaginings; until, by its removal, consciousness returns, which is always in a few moments after she ceases to inhale, when she often weeps that 'she has been brought back to earth again.'*

* The writer had a case of this kind of peculiar interest in his own practice a short time since, in the person of a lady, under the influence of chloroform, for the extirpation of malignant hemorrhoids, in which it was with the utmost difficulty she could be composed when she found she was really yet on earth—and many such instances are known to us.

There is generally during the first two or three inhalations an increased rapidity of the respiration, also of the pulse; but these, soon becoming slow and regular, like natural sleep, the patient passes almost imperceptibly into the land of glorious visions, where, for aught we know, she revels amid the homes of the blessed, and holds converse with the spirits that surround the throne. Different individuals or constitutions manifest different degrees of quiet or excitement when under the influence of this agent. While one becomes perfectly calm and composed after the first two or three inhalations, another will be restless, tossing about, and manifesting signs of suffering; but awake her, and ask her if she suffers pain, and she will tell you 'No. Give me more chloroform.'

The frail and delicate female will often remain conscious and converse with perfect freedom during the intervals of pain, and, on the slightest indications of its return, apply the sponge saturated with chloroform to her mouth; and, thus, while under the most powerful uterine contraction, free herself from all consciousness of suffering. Often, when restless and talkative, a little increase of the chloroform will quiet all in sound sleep. We have often seen her, while sufficiently under its influence to destroy all sensation of pain, chat freely and answer questions correctly. We have seen the mother, in the last stages of labor perfectly quiet under the most intense uterine pressure. We have seen her when, for thirty-two hours, her whole frame had been in an agony of torture, relieved of her sufferings, the instruments applied, and delivery accomplished in ten or fifteen minutes from the time of its first inhalation. We have seen the attached placenta carefully removed, while the unconscious patient was reveling amid the sunny scenes of her girlish days, or, with buoyant spirits, waiting in her elysian world for the first fond accents that should pronounce her mother. We have seen her pass through almost the entire course of labor without the slightest sufferings, and awake to find herself a happy parent, and 'bless Heaven for chloroform.'

But it is objected, 'That the patient suffers just as much as though she were not under its influence, but is not conscious of it.' Now these manifestations are easily accounted for on perfectly rational principles; as in sleep, for instance, when for some cause or other a function is disturbed, the brain takes cognizance of this disturbance, and efforts are made until a change is effected, without the least consciousness on the part of the individual. The person may *will* to turn himself, but perceives it not.

The motory and sensory nerves, we know, have their origin in different columns of the spinal cord; and in the organs of organic life we find chloroform selecting, as it were, which of these it will suspend and which it will leave untouched. The functions it would control are selected and held under its mysterious influence, while others are left to pursue their natural course. Suffering, we think, must consist in a consciousness of pain: hence, where there is no consciousness there can be no suffering.

Again, it is urged, that 'Anæsthesia suspends uterine contraction, and consequently retards labor.' But experience and *facts* both abundantly prove, that, when rightly administered, that is not the result of chloroform.

But here are some of its effects (and we speak that we do know):—At the commencement of labor, when the pains have been frequent and harrassing, without seeming to advance the stage of labor, but only wearying the patient, a small quantity of chloroform inhaled will produce sleep; the patient sinks into a quiet repose for fifteen or thirty minutes, during which nature rallies, the mother is refreshed, and labor proceeds unmolested to its close. All this simply proves, that, whereas, the contractions were frequent, 'vexing,' and inefficient, they now become regular, *efficient*, and painless. It is a frequent occurrence, as every one knows who has been in the habit of using chloroform, that from the most unpromising and painful, the most speedy and favorable labors have occurred. We do not contend that this agent might not be pushed so far as to suspend uterine contraction altogether. So it *might*, to suspend even *life;* but this, in parturition, need not be so: the judicious practitioner will, in every case, avoid it.

We hear it also objected, that 'The law of succession should prevent our using chloroform in child-birth, as it may, ere we are aware, reach the medulla oblongta, and thus destroy life.' Now it is a fact that this process of 'succession' never can be understood, since its effects are so sudden, often not requiring more than one or two inhalations to produce the result. But shall we, therefore, abandon its use? Every principle in in science, not self-evident, is proven to be such by actual experiment. And shall we abandon a great truth because we find, or cannot account for one exception in a thousand? Suppose we should *certainly* lose one in a thousand, would this argue that the nine hundred and ninety-nine were based upon mere chance? that the untold sufferings that had been allayed in all the other cases were the result of mere accident? Would we not rather, and justly too, attribute it to some other or unknown cause, something may-be yet beyond the depths of our present knowledge? If we adopt this method of reasoning, we destroy the value of statistics, which, in establishing facts and principles, are of the utmost importance. If this be true, it matters not if we had a countless array of this kind of proof, one single exception would destroy the whole.

But we contend we *have* a right to draw conclusions from actual experiments, and to *experiment* when we are satisfied that, on the whole, more of *good* than of *evil* will result from our experiment.

It is objected also, that 'The decree has gone forth, "In sorrow shalt thou bring forth,"' and that, therefore, we are interfering with the designs of Providence to seek to abolish pain in child-bearing. Upon the same principle we might object to administering any agent for the relief of pain or disease, as disease comes upon us as part of the curse for sin. Or, still further, we might object to the manufacture or use of *labor-saving* machines, because the curse has gone out, 'In the *sweat* of thy brow shalt thou earn thy bread.'

And still, again, says the objector, 'The pains of child-bearing are really too trifling to demand the use of so potent an agent, as they are purely *functional*.' But we contend that there is no such thing as functional pain in parturition. Pain is *not* the result of uterine contraction, but of the impediments to the passage of the fœtus, such as the unyielding state of the fœtal head, narrowness of the passage, etc. and that, therefore, anæsthesia does not disturb functional harmony. But who shall be authority, whether the pains of child-birth are really 'trifling'? Certainly not he who makes this assertion. The proof must come from the actual sufferer; and all writers agree upon the testimony, the oft-repeated and *soul-stirring* testimony, that the pains of labor are *ever painful*, and often *severely* so. Who that has ever witnessed that look of anguish, those contortions of the frame; those deep, agonizing groans, not to be described; can deny this truth? Who has not often felt that he would be willing to make almost any sacrifice in his power to allay these sufferings? We verily believe the hour has come when the question to be decided is, not whether the suffering is great and demanding our sympathy; but whether, before Heaven and suffering humanity, we shall be exculpable in withholding this agent when it is admissable to use it.

Says Prof. Simpson, 'Instead of determining in relation to it (natural labor), whether we shall be justifiable in using this agent under the circumstances named, it will become, on the other hand, necessary to determine whether, on any grounds, moral, or medical, or professional, man could deem himself justifiable in withholding and not using any such means (as we suppose this to be); provided he had the power of assuaging the agonies of the last stage of labor:—and we might add, counteracting what Velpeau describes, as 'those piercing cries, that agitation so lively, those excessive efforts, those inexpressible agonies, and those pains apparently intolerable, which accompany the natural parturition of the human mother.'

This means has, then, in the progress of human science, been brought to light. We have it in our power to assuage every pain of parturition, and to cause what was once more dreaded than *death* itself, now to be looked upon as but one of the changes in life which go to make up the sum of human happiness.

Are we asked, 'Would you administer chloroform in every case of natural labor?' We answer, *Yes!* wherever we could prevent but a few minutes of suffering, provided no organic impediment or constitutional derangement stood in the way, such as disease of the heart from dropsy, induration, thickening or leakage of the valves from any cause, or some serious weakness or determination to the brain. Mere neuralgia or nervous debility of the heart, or asthmatic affections of the lungs, would not prevent our giving it. Indeed, we have often given it to the confirmed consumptive with the best of results. We have administered it by mouth when the patient was sinking by suffocation from 'enlargement of the heart,' when nothing else would give relief.

As to the precise time in labor when we would commence the use of chloroform, it would be difficult to give any fixed rule. Every practitioner must be governed by the circumstances of the case and his own good judgment, and *experience* will best give him confidence in determining him in its administration. We often give it quite early, when the pains have been, as they are often termed, 'vexing,' 'wrangling,' or 'wearying,' that quiet and composure of the nervous system may be brought about, and thus, after a little rest, nature is better prepared to accomplish its object. As a general thing, we put the patient under its influence, when the os is dilated or dilating, and the pains have become regular and well developed. Indeed, we have often seen a protracted rigidity of the os yield to a few moments' sleep from the chloroform, and a hitherto *tedious* labor become easy and efficient. We have found no means so free from objections in giving the chloroform, as a simple sponge hollowed out to fit the mouth and nose. Upon this (after having been moistened and thoroughly wrung out of warm water) we pour a drachm, more or less, of pure chloroform, always managing the sponge ourselves, until our patient comes under its influence. We graduate the quantity *ourselves*, according to the circumstances of the case, and never trust another to pour the chloroform without our inspection. When the patient comes under its control, we commit the sponge to the care of some judicious lady present, giving directions when to apply and when not, always seeing that *plenty of fresh air is inhaled with it.* The sponge is held at a short distance from the nose for the first two or three inhalations, to prevent strangling, and then the sooner the patient comes under its influence the better. We have often seen it administered in small quantities and slowly, thus producing merely excitement and fruitless attempts to anæsthetize the patient, thus bringing disrepute upon the practitioner and the chloroform, when a little more familiarity with and confidence in the powers of this agent would have induced him to push it to the desired effect at once, or, by reasoning with his patient, encouraged her to inspire it freely, and with confidence compose herself to its influence. It is not necessary to push it to the same extent in parturition that it must be in surgery, hence its greater safety. By two or three inhalations, or in a very few moments, your patient will become completely under its influence, and thus your object be gained. Should she become restless and talkative, this may be quieted by pushing the chloroform a little further, or if sensation of suffering is destroyed the talking matters but little. Thus we have kept patients under its influence for from fifteen minutes to fourteen hours, graduating the quantity according to the *indications.* We have seen, in the books, that ergot must not be given if you would administer chloroform; but we give both together, whenever we deem it proper, and always with good results. Unless in *instrumental* labor, it is not necessary to push chloroform to stertorous breathing, but in these cases we have seen it manifest its wondrous power in controlling pain. The best index as to the real condition of your patient, is the state of the respiration. We pay but little attention to the pulse, provided the breathing is regular.

In cases of *death* from chloroform, the respiratory functions cease before the cardiac; the heart continuing its action uninfluenced by the chloroform for a period longer or shorter after the respiratory has ceased. Its then ceasing may be considered as a natural consequence of the lack of the respiratory action of the lungs, and as independent of the use of chloroform. But if the respiratory has failed, and while the heart is still in action the chloroform be continued, the movements of the heart will then become impaired, and its cessation may be justly attributed to the effects of this agent. From what we have heard and read on this subject, we are confident that much time is lost and injury done in attempts to resuscitate patients sinking under the influence of chloroform, by applying stimulants to the nostrils or attempting to pour cordials into the stomach when the respiratory functions have ceased; for, in this case, the former can do no good, and the latter would be as likely to pass into the trachea and bronchi as into the stomach. Says a writer for the *Edinburgh Monthly Journal:*—'When the patient is lying, so soon as the breathing ceases and the jaw drops, the tongue is particularly liable to fall backwards and fill the orifice of the glottis. Artificial respiration under such circumstances is worse than useless. It is better at once to pull the tongue well out of the mouth, and passing a hook through the tip confide it to the care of an assistant.' He says further:—'I am convinced that in some cases in which respiration has ceased, it has been from neglect or too tardy adoption of this simple method.' But we are happy to say, that, in the use of chloroform, in our own practice, no such means have ever been necessary, nor do we believe such results *ever* necessary in its administration in child-birth.

We cannot say that we are aware of *one* well-authenticated case on record of death in natural labor from the use of chloroform. We have seen a notice, from the *Medical Times and Gazette*, of April, 1855, recording an instance of 'death from chloroform administered by a nurse.' But the condition of the patient at the time, or the qualifications of the nurse to administer the agent, we know nothing of: for aught we know, the same nurse, at the same time, might as well have poisoned her patient with opium, spirits turpentine, lobelia, or any poisonous narcotic. Some time since we saw a newspaper report of 'Death from chloroform in child-birth.' It is possible chloroform had as much to do with the death in this instance as it had in producing 'ague' in a case to which we were once called. The lady had intended taking chloroform in her confinement, but the labor having terminated before my arrival, she of course did not take it. Some weeks after she was sick from 'ague,' and the report went the rounds that 'she had never rallied from the effects of the chloroform which the Doctor gave her.' On investigation we have found many of these reported 'deaths' based upon about as reasonable grounds.

But suppose *one* should die of the thousands who inhale chloroform, or even if it were *certain* that *one* in every thousand who take it should die, should this deter us entirely from giving it to the hundreds of thousands of suffering multitudes, *many* of whom *must* die unless relieved from suffering by its power? Professor Simpson has reported some three thousand cases of child-birth under the influence of chloroform, and without any unfavorable results in a single case, and hundreds of thousands more have taken it, and blessed Heaven for it. It is only strange, that in a period of over nine years since its first use, but *one* case of death should have occurred to the parturient, and this is a very doubtful one. But we have passed the day when an exception like this, or the few that may have occurred, should deter us from its administration, wherever, by its proper use, we can allay the tortures of pain or stay the ravages of disease.

